# Structure of the Varicella Zoster Virus Thymidylate Synthase Establishes Functional and Structural Similarities as the Human Enzyme and Potentiates Itself as a Target of Brivudine

**DOI:** 10.1371/journal.pone.0143947

**Published:** 2015-12-02

**Authors:** Kelly Hew, Sue-Li Dahlroth, Saranya Veerappan, Lucy Xin Pan, Tobias Cornvik, Pär Nordlund

**Affiliations:** 1 Division of Structural Biology and Biochemistry, Nanyang Technological University, School of Biological Sciences, Singapore, Singapore; 2 Division of Biophysics, Department of Medical Biochemistry and Biophysics, Karolinska Institutet, Stockholm, Sweden; 3 Institute of Molecular and Cell Biology, Agency for Science, Technology and Research, Singapore, Singapore; Monash University, AUSTRALIA

## Abstract

Varicella zoster virus (VZV) is a highly infectious human herpesvirus that is the causative agent for chicken pox and shingles. VZV encodes a functional thymidylate synthase (TS), which is the sole enzyme that produces dTMP from dUMP *de novo*. To study substrate binding, the complex structure of TS_VZV_ with dUMP was determined to a resolution of 2.9 Å. In the absence of a folate co-substrate, dUMP binds in the conserved TS active site and is coordinated similarly as in the human encoded TS (TS_HS_) in an open conformation. The interactions between TS_VZV_ with dUMP and a cofactor analog, raltitrexed, were also studied using differential scanning fluorimetry (DSF), suggesting that TS_VZV_ binds dUMP and raltitrexed in a sequential binding mode like other TS. The DSF also revealed interactions between TS_VZV_ and *in vitro* phosphorylated brivudine (BVDU_P_), a highly potent anti-herpesvirus drug against VZV infections. The binding of BVDU_P_ to TS_VZV_ was further confirmed by the complex structure of TS_VZV_ and BVDU_P_ solved at a resolution of 2.9 Å. BVDU_P_ binds similarly as dUMP in the TS_HS_ but it induces a closed conformation of the active site. The structure supports that the 5-bromovinyl substituent on BVDU_P_ is likely to inhibit TS_VZV_ by preventing the transfer of a methylene group from its cofactor and the subsequent formation of dTMP. The interactions between TS_VZV_ and BVDU_P_ are consistent with that TS_VZV_ is indeed a target of brivudine *in vivo*. The work also provided the structural basis for rational design of more specific TS_VZV_ inhibitors.

## Introduction

Varicella zoster virus (VZV) is a highly contagious human herpesvirus that causes varicella, which is commonly known as chicken pox [1]. Like all other human herpesviruses, VZV infection is life-long and the virus establishes its latency in dorsal root ganglia [2, 3]. Reactivation of VZV from latency can cause herpes zoster or shingles, which occurs more frequently in older adults and immunocompromised individuals [1]. The herpes zoster lesions have been associated with acute and persisting pain that may last beyond a month after the initial outbreak [4].

Herpesviral DNA synthesis is pivotal for the production of new infectious virus particles in the host cells. This is governed by activities of DNA synthesis proteins, which have been the focus in the development of many anti-herpesvirus drugs [5, 6]. A large proportion of the clinically approved anti-herpesvirus drugs are nucleoside analogs that have inhibitory actions against the viral DNA polymerase upon their conversions to the nucleotide triphosphosphates [7, 8]. These include the acyclic nucleoside analogs aciclovir, valaciclovir, penciclovir, famiciclovir, ganciclovir and valgancyclovir, the deoxycytidine analog cidofovir, and the 5-substituted pyrimidine nucleoside analogs idoxuridine (IDU) and brivudine (BVDU) [7, 8]. The safety and selectivity of most of these nucleoside analogs are dependent on the first phosphorylation step by the viral thymidine kinase (TK) that is only encoded in a virally infected cell [9, 10].

Thymidylate synthase (TS) is a highly conserved protein found through all kingdoms of life including human, mouse, bacteria, protozoa and some viruses. It catalyzes the transfer of the methylene group from cofactor 5,10-methylenetetrahydrofolate (mTHF) to substrate deoxyuridine monophosphate (dUMP) [11]. In this process, deoxythymidine monophosphate (dTMP) is produced *de novo* and mTHF is converted to dihydrofolate (DHF). Subsequently, dTMP undergoes another two successive phosphorylation steps to produce deoxythymidine triphosphate (dTTP), which is added to the elongating DNA chain by the DNA polymerase [11].

Due to its essential role in the DNA replication, the human TS (TS_HS_) has been extensively studied and is a well-proven drug target for cancer therapy [12]. Inhibition of TS_HS_ has been shown to lead to a deficiency in dTTP and DNA damage due to insertion of deoxyuridine triphosphate (dUTP) into DNA. This results in a cell death, so called thymineless death [13]. Different TS_HS_ inhibitors have been derived from modifications of its natural substrate dUMP and cofactor mTHF. These include the nucleobase analog 5-fluorouridine and the folate analogs raltitrexed and pemetrexed, which are currently used in treatments against solid tumors [14–16]. Molecular features of TS_HS_ and other TS have been elucidated in complex structures with different substrates and/or inhibitors [17–30]. These structures have contributed greatly to rational structure based drug designs that improved the specificity and efficacy of TS_HS_ inhibitors [31].

For reasons not completely clear, only VZV and Kaposi’s sarcoma associated herpesvirus (KSHV) encode a TS (TS_VZV_ and TS_KSHV_ respectively) [32, 33]. Both TS_VZV_ and TS_KSHV_ share more than 50% sequence identity with TS_HS_ but unlike the well-studied human counterpart, the homologous herpesviral TSs remain poorly characterized and no structural information is available on these proteins. TS_VZV_ has also been shown to be non-essential for the viral replication *in vitro* [34]. In an attempt to gain insights into TS_VZV_ and to differentiate TS_VZV_ from TS_HS_, biophysical analysis and structural analyzes of TS_VZV_ with its substrate dUMP and a TS_HS_ inhibitor raltitrexed were carried out. The structures of TS_VZV_ and TS_VZV_ in complex with dUMP were solved to a resolution of 3.1 Å and 2.9 Å respectively. This constitutes the first crystal structure of an eukaryotic virus TS. The biophysical assays and the complex structure of TS_VZV_ with dUMP demonstrated similar modes of substrate binding for TS_VZV_ and TS_HS_. Structural comparisons of TS_VZV_ and TS_HS_ also revealed similar substrate binding details but with slight amino acids variations in the TS active site. Biophysical and crystallographic studies of TS_VZV_ with an *in vitro* phosphorylated BVDU supports binding of phosphorylated BVDU to TS_VZV_. This study also provided further support for that TS_VZV_ is a potential target enzyme for BVDU after metabolic activation in infected cells.

## Results

### Structure of apo TS_VZV_

The structure of the apo TS_VZV_ (apo-TS_VZV_) was determined and refined to a final resolution of 3.1 Å, with four molecules in each asymmetric unit (Table 1 and S1 Fig). Apo-TS_VZV_ was crystallized with a protein construct comprising of amino acids 8 to 295, which lacks the N-terminus seven amino acids and C-terminus six amino acids. This model has electron densities for amino acids 15–295, except for a missing loop containing amino acids 38–39 in one of the four molecules in the asymmetric unit. The four TS_VZV_ monomers in the asymmetric unit are arranged as two homo-dimers. The monomers are virtually the same and the Cα atoms superimpose with a root mean square deviation (rmsd) of 0.78 Å.

**Table 1:** Data collection and refinement statistics on the apo and complex structures of TS_VZV_.

	Apo TS_VZV_ (4XSE)	TS_VZV_+dUMP (4XSD)	TS_VZV_+BVDU_P_ (4XSC)
**Data Collection**			
Beamline	AS MX1	AS MX1	AS MX1
Wavelength (Å)	0.9537	0.9537	0.9537
Resolution Range (Å)	29.7–3.1 (3.1–3.2)^a^	30.0–2.9 (2.9–3.0)^b^	30.0–2.9 (2.9–3.0)^b^
Space group	p3_2_	p3_2_	p3_2_
Unit cell	153.4 Å 153.4 Å 89.2 Å	150.2 Å 150.2 Å 89.2 Å	149.5 Å 149.5 Å 89.0 Å
	90.0° 90.0° 120.0°	90.0° 90.0° 120.0°	90.0° 90.0° 120.0°
Total reflections	155476	1195011	94756
Unique reflections	42243	49612	46238
I/I(σ)	8.5 (2.4)^a^	19.8 (3.9)^b^	8.1 (1.9)^b^
Multiplicity	3.7 (3.4)^a^	5.5 (5.2)^b^	1.5 (1.5)^b^
Completeness (%)	99.2 (97.5)^a^	100.0 (99.9)^b^	96.2 (95.3)^b^
R_merge_ ^c^ (%)	12.7 (47.5)^a^	6.9 (38.0)^b^	9.7 (40.3)^b^
**Refinement**			
R_factor_ ^d^ (%)	23.8	24.0	19.6
R_free_ ^e^ (%)	26.8	28.0	23.5
Protein residues	1116	1081	1244
Solvent	0	9	30
Ligands	4 phosphate ions	4 dUMP	4 BVDU_P_
			4 1PE
RMSD bonds (Å)	0.5	0.0045	0.002
RMSD angles (°)	1.134	0.882	0.585
Ramachandran favored (%)	95	96	94.4
Ramachandran allowed (%)	5	4	5.1
Ramachandran outliers (%)	0	0	0.5
Rotamer outliers (%)	4.4	6.4	0.8

^a^ The values in the parentheses are for the highest resolution shell (3.1–3.2 Å).

^b^ The values in the parentheses are for the highest resolution shell (2.9–3.0 Å).

^c^ $R_{merge}=\frac{\left[\sum_{hkl}\sum_{i}\left|I_{i}\left(hkl\right)-\bar{I}\left(hkl\right)\right|\right]}{\left[\sum_{hkl}\sum_{i}\bar{I}\left(hkl\right)\right]}\times 100$, where *I*_*i*_ is the *i*th intensity measurement of reflection *hkl*, *Ī(hkl)* is the mean intensity measurement of the symmetry related or replicated reflections of the unique reflection *hkl*.

^d^ $R_{factor}=\frac{\left[\sum_{hkl}\left|F_{obs}\left(hkl\right)-F_{calc}\left(hkl\right)\right|\right]}{\left[\sum_{hkl}F_{obs}\left(hkl\right)\right]}\times 100$, where *F*_*obs*_ and *F*_*calc*_ are the observed and calculated structure factors respectively.

^e^ R_free_ is equivalent to R_factor_ but 5% of the measured reflections have been excluded from refinement and set aside for cross validation.

**Fig 1:**
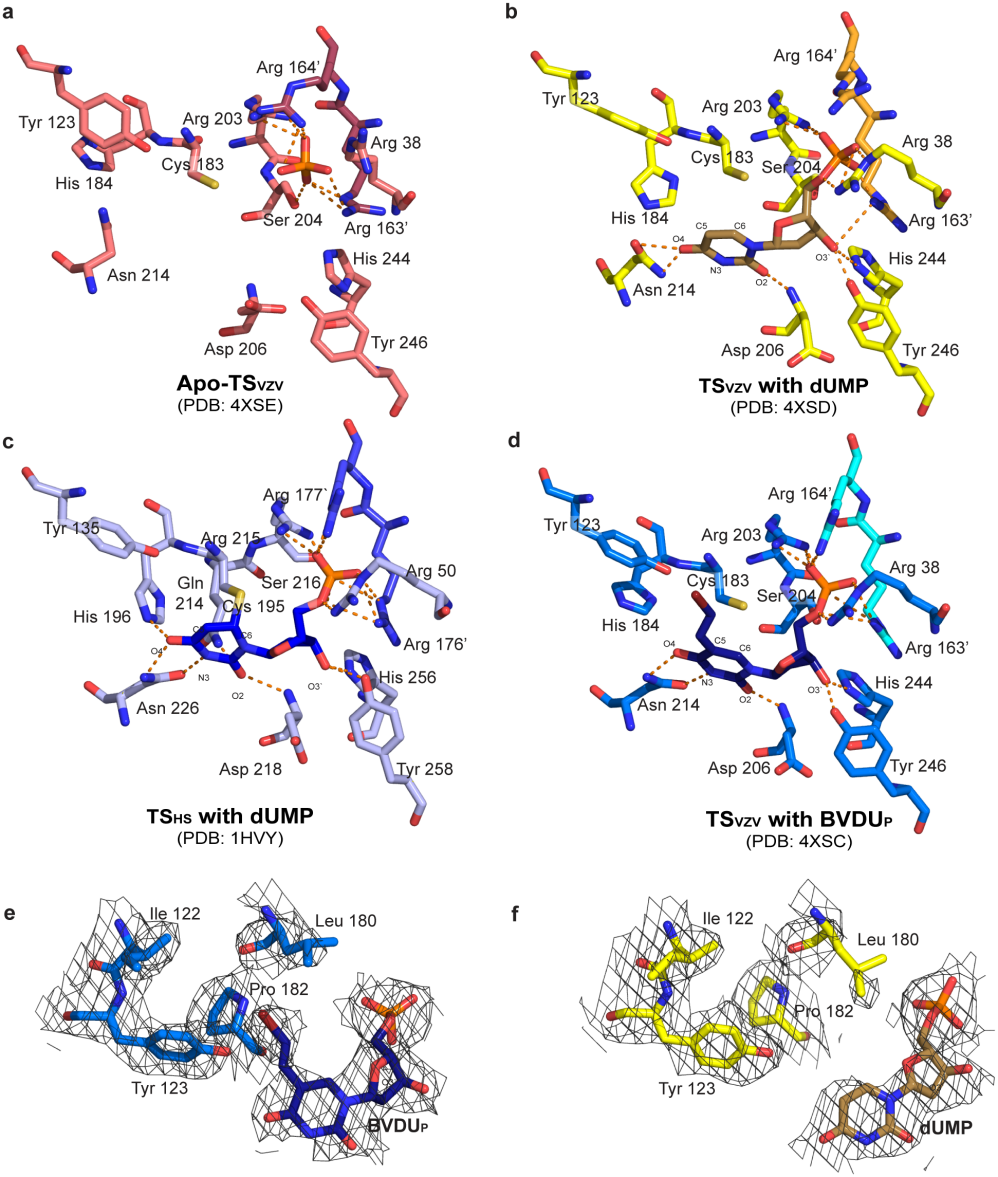
Stereo views of the TS_VZV_ and TS_HS_ active sites. Corresponding stereo views of the ligands and amino acids lining the TS active sites in the structures of (a) apo-TS_VZV_ with a phosphate ion, (b) TS_VZV_ with dUMP, (c) TS_HS_ with dUMP (PDB ID: 1HVY) [35] and (d) TS_VZV_ with BVDU_P_. The polar interactions between the amino acids and the different ligands are illustrated by orange dotted lines. 2F_0_-F_C_ electron densities of the binding (e) BVDU_P_ and (f) dUMP with the surrounding hydrophobic amino acids are also shown (contoured at 1σ with carved = 2.0).

The structures of apo-TS_VZV_ and the human encoded apo TS (TS_HS_) superimpose with a rmsd of 0.9 Å for 558 residues, with the highest similarity at the active sites [22]. A phosphate ion was found at one end of the active site in each of the TS_VZV_ monomers and is held in place by Arg 203, Ser 204, as well as Arg 163’ and Arg 164’ that are located on the loop from the dimer partner (Fig 1a).

### Complex structure of TS_VZV_ with dUMP

TS_VZV_ was crystallized in a second crystal form that was used for subsequent determination of the structure of TS_VZV_ in complex with dUMP (TS_VZV_+dUMP). The TS_VZV_+dUMP structure was determined and refined to a resolution of 2.9 Å (Table 1). Each asymmetric unit contains four TS_VZV_ subunits arranged into two homo-dimers. Each subunit is made up of amino acids 15 to 295. This is with exception to amino acids 98 to 117 in one monomer of the TS_VZV_ dimer, and amino acids 38 to 39 and 136 to 139 in the other monomer of the TS_VZV_ dimer. Electron densities in these regions are not continuous and could therefore not be modeled.

A molecule of dUMP binds in the TS_VZV_ active site, where the phosphate group is coordinated by Arg 38, Arg 203, and Arg 163’ of the dimer partner (Fig 1b). O3’ of the pentose sugar group makes a hydrogen bond to the hydroxyl group of Tyr 246 and the pyrimidine ring forms hydrogen bonds with Asp 206 and Asn 214 (Fig 1b). O2 interacts with the main chain amide group of Asp 206 while O4 binds Nδ1 and Oδ1 of Asn 214.

Comparison of the apo-TS_VZV_ and TS_VZV_+dUMP structures reveals no major conformational difference upon the binding of dUMP (Fig 1a and 1b). Cα backbones of TS_VZV_ monomers in both structures overlay well with an average rmsd of 0.69 Å. Only small side chain rearrangements are seen for the amino acids lining the active site. The most noticeable changes are observed for amino acids interacting directly with dUMP (Fig 1a and 1b). In the presence of dUMP, the side chains of Asp 206, Asn 214 and Tyr 246 shifted for direct interactions with the pentose sugar and pyrimidine ring. The loop spanning amino acids 204 to 206 also inclined towards dUMP for direct interactions. The side chain configurations of Arg 163’ and Arg 164’ from the other monomer are also slightly displaced. Although Tyr 123 does not make direct interactions with dUMP, the tyrosine ring is rotated in the presence of dUMP.

### Structural comparison of the coordination of dUMP in TS_VZV_ and TS_HS_

Complex structures of different TS have been described to exist in either an open or a closed conformation [22, 35]. Both conformations are structurally conserved but the thiol group of the catalytic cysteine in the closed conformation is in an active complex and binds covalently with C6 of the dUMP pyrimidine ring [22, 36]. The catalytic cysteine corresponds to Cys 183 in TS_VZV_ or Cys 195 in TS_HS_ (Fig 1b and 1c). The structure of TS_VZV_+dUMP superimposes well with the open and closed conformations of TS_HS_ with an average rmsd of 0.7 Å and 0.9 Å along the Cα backbone respectively.

The coordination of dUMP in the active site of TS_VZV_ was compared with the closed TS_HS_, since the latter was reported to represent the conformation of a catalytically active TS_HS_ [22]. The overall coordination of dUMP in the active sites of both TS_VZV_ and TS_HS_ is highly similar with slight differences. The dUMP phosphate group is coordinated by three conserved arginines (Arg 38, Arg 203, and Arg 164’) in TS_VZV_ but four conserved arginines (Arg 50, Arg 215, Arg 176’ and Arg 177’) in TS_HS_ (Fig 1b and 1c). O2 of the dUMP pyrimidine ring binds to the main chain amide of Asp 206 in TS_VZV_ and the corresponding Asp 218 in TS_HS_. Both Asn 214 in TS_VZV_ and the corresponding Asn 226 in TS_HS_ bind to O4 of the dUMP pyrimidine ring.

Differences are also observed in the coordination of dUMP in TS_VZV_ and TS_HS_ (Fig 1b and 1c). While the catalytic cysteine is conserved in TS_VZV_, it does not appear to be covalently bound to dUMP as in some analog complexes from other TS (Fig 1b) [22, 36]. Distance of the catalytic cysteine thiol group to C6 of the dUMP binding to TS_HS_ in the closed TS_HS_ structure (PDB ID: 1HVY) was reported to be 2.1 Å [22]. The distance between the thiol group of the corresponding Cys 183 and C6 of dUMP in the TS_VZV_+dUMP structure varies across the four TS_VZV_ subunits in the asymmetric unit, ranging from 3.2 Å to 3.7 Å and averages at 3.4 Å. This is more similar to the structure of TS_HS_ in complex with only dUMP (PDB ID: 3HB8), where the distance between the catalytic thiol and C6 of the dUMP is also approximately 3.4 Å. This is consistent with the proposal that folate is required for formation of the covalent bond between dUMP and the catalytic cysteine [35]. In addition, O3’ of the dUMP pentose sugar group is hydrogen-bonded to His 246 in TS_VZV_ and the corresponding His 258 in TS_HS_. However, O3’ is also hydrogen-bonded to Tyr 256 in TS_HS_ but not the corresponding Tyr 244 in TS_VZV_. The distance between O3’ and NE2 on Tyr 244 in TS_VZV_ is 3.5 Å, which is too far for Tyr 244 to be a hydrogen bond donor. An additional hydrogen bond also exists between N3 of the pyrimidine ring and Oδ1 of Asn 226 in TS_HS_ but not between N3 and the corresponding Asn 214 in TS_VZV_. Another distinctive difference is located at His 184 (His 196 in TS_HS_). In TS_HS_, there exists a direct interaction between the imidazole ring of His 196 and O4 of the pyrimidine ring but in TS_VZV_, the corresponding His 184 is tilted and does not make any direct interactions with dUMP (Fig 1c and 1d).

### Structure of TS_VZV_ in complex with BVDU_P_

The structure of TS_VZV_ in complex BVDU_P_ was also determined by soaking BVDU_P_ with crystals of the second crystal form. The complex structure was determined and refined to a resolution of 2.9 Å (Table 1). There are also four TS_VZV_ monomers that are arranged into two homo-dimers in each asymmetric unit. Each monomer is made up of amino acids 15 to 295, except the disordered amino acids 98 to 117 in one monomer of the TS_VZV_ dimer.

The active site of each monomer contains a molecule of BVDU_P_ (Fig 1d). However, there is no electron density for the bromide atom in two of the four subunits in the asymmetric unit. The missing bromide atom in BVDU_P_ is likely attributed to radiation damage during data collection [37, 38]. A molecule of PEG 400, that is likely to be contributed by the crystallization buffer, is also found in the active site. It binds to BVDU_P_ and is located in the TS_VZV_ active site where the folate binds.

The BVDU_P_ phosphate group is coordinated by the conserved Arg 38, Arg 203 and Ser 204 from one monomer, and Arg 163’ and Arg 164’ from the other monomer (Fig 1d). The pentose sugar on BVDU_P_ interacts with the surrounding residues through hydrogen bonds involving O3’. O3’ is hydrogen-bonded to both the hydroxyl group on Tyr 246 and Nε2 on the imidazole ring of His 244. BVDU_P_ is also held in place through interactions involving the pyrimidine ring. O2 of the BVDU_P_ pyrimidine ring is hydrogen-bonded to the main chain amide of Asp 206. O4 and N3 of the BVDU_P_ pyrimidine ring are also hydrogen-bonded to Nδ1 and Oδ1 of Asn 214 respectively. The 5-bromovinyl substituent on BVDU_P_ stretches towards and binds the hydrophobic patch on the active site that is lined by amino acids Tyr 100, Ile 122, Tyr 123, Leu 180 and Pro 182 (Fig 1e).

No major conformational difference is observed between the structures of TS_VZV_+BVDU_P_ and TS_VZV_+dUMP. However, the position and coordination of BVDU_P_ deviate somewhat from dUMP in the active site of TS_VZV_. As a result, the direct interactions of BVDU_P_ and TS_VZV_ is not conserved with that observed between dUMP and TS_VZV_ (Fig 1b and 1d). The O3’ of the pentose sugar in BVDU_P_ is hydrogen-bonded to His 244 and Tyr 246 but that in dUMP only interacts with Tyr 246. An additional interaction also exists between N3 of the BVDU_P_ pyrimidine ring and Oδ1 of Asn 214. The distance between the thiol group atom on Cys 183 to C6 of the pyrimidine ring in dUMP and BVDU_P_ decreased from 3.4 Å to 3.0 Å respectively. The 5-bromovinyl substituent in BVDU_P_ also caused slight side chain perturbations at Tyr 100, Ile 122, Tyr 123, Leu 180 and Pro 182 (Fig 1e and 1f).

In contrast, the coordination of BVDU_P_ in TS_VZV_+BVDU_P_ is more similar with the coordination of dUMP in the closed-TS_HS_ structure (PDB ID: 1HVY) [22] (Fig 1c and 1d). The coordination of the phosphate groups, pentose sugars and pyrimidine rings of both BVDU_P_ and dUMP is highly conserved in both TS_VZV_ and TS_HS_. This is with exception to His 184. In TS_HS_, O4 of the dUMP pyrimidine ring is hydrogen-bonded to Nε2 of His 196 but in TS_VZV_, the corresponding His 184 is not hydrogen-bonded to O4 of the BVDU_P_ pyrimidine ring. The imidazole ring of His 184 in three of the four TS_VZV_ subunits in the asymmetric unit is rotated approximately 50–60° about Cβ from the corresponding His 196 in TS_HS_ and does not bind to O4 of the BVDU_P_ pyrimidine ring directly. Even though the conformation of His 184 in one of the TS_VZV_ subunits in the asymmetric unit is more similar to the corresponding His 196 in TS_HS_, the distance between His 184 and BVDU_P_ is still too far for direct interaction.

### Binding studies of TS_VZV_ using differential scanning fluorimetry (DSF)

**Fig 2:**
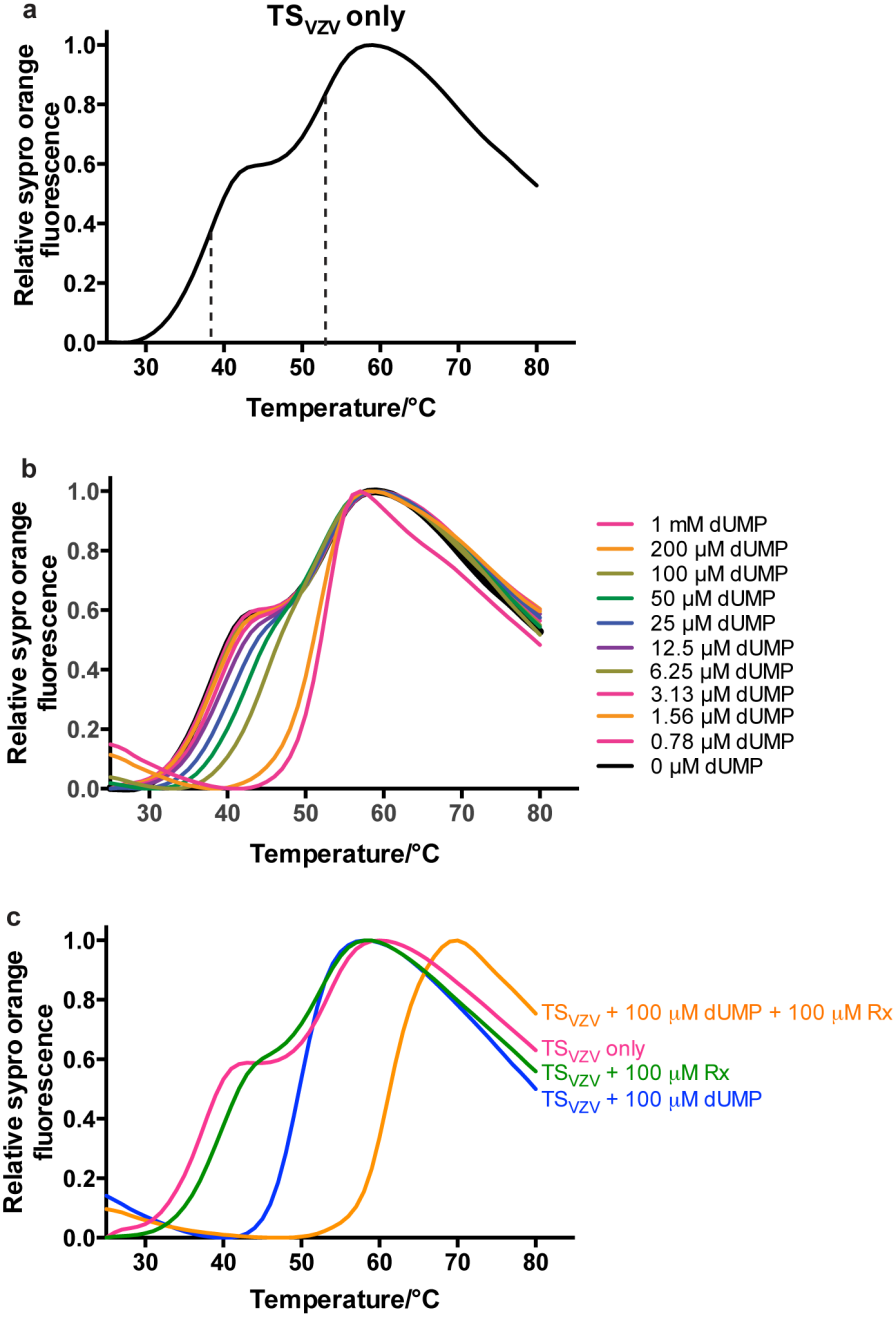
DSF melting curves of TS_VZV_ with dUMP. (a) The DSF melting curve of apo-TS_VZV_ has two melting transitions and two T_m_ can be estimated from the mid-slope of each sigmoidal transition. Dotted lines were added to the mid-slopes of each melting curve as references to the TS_VZV_ T_m_ for each melting transition. (b) Increasing concentrations of dUMP only increased the T_m_ of the first melting transition. 100 μM of dUMP was sufficient to stabilize the first melting transition of TS_VZV_ to produce a single melting transition. Further increase of the dUMP concentration could not increase the T_m_ beyond the second melting transition. (c) Raltitrexed further stabilized TS_VZV_ in the presence of 100 μM of dUMP in the DSF. In the absence of dUMP, raltitrexed could not stabilize TS_VZV_ in DSF.

To study the binding of ligands to TS_VZV_, we explored a thermal stability shift assay based on differential scanning fluorimetry (DSF). In the absence of any ligand, DSF shows that TS_VZV_ has a dual-transition melting curve that is made up of two sigmoidal curves (Fig 2a). The melting temperature (T_m_) of the first and second melting transition of TS_VZV_ in the absence of any ligand were estimated to be 38°C and 53°C respectively (Fig 2a). When TS_VZV_ was titrated with different concentrations of dUMP, a shift of the first transition of the TS_VZV_ melting curve was seen, while the second melting transition remained unchanged (Fig 2b). The first transition eventually disappeared with 100 μM of dUMP and further increase of the dUMP concentration could not increase the T_m_ beyond the second melting transition (Fig 2b).

The T_m_ of TS_VZV_ is 50°C in the presence of 100 μM dUMP (Fig 2c). When 100 μM raltitrexed and 100 μM dUMP was added to TS_VZV_, the T_m_ of TS_VZV_ increased from 50°C to 61°C (Fig 2c). In the absence of dUMP, the addition of 100 μM raltitrexed produced a dual-transition melting curve that is largely similar to the melting curve of TS_VZV_ without any ligand (Fig 2c). No significant stabilization effect on the first and second melting transitions was observed with raltitrexed (Fig 2c). This is consistent with the notion where dUMP must be bound in order for the successive binding of raltitrexed in the active site of TS_HS_ [18].

**Fig 3:**
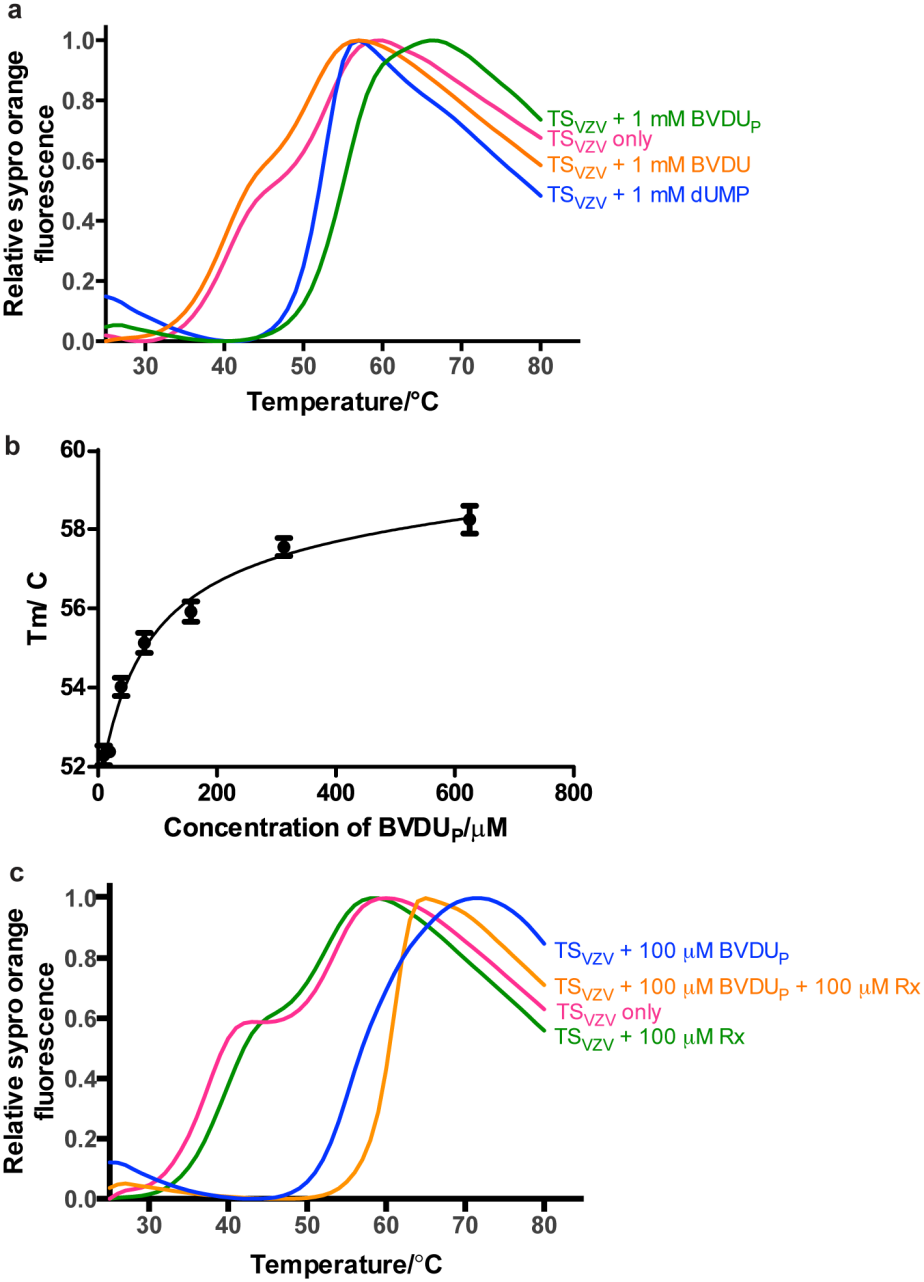
DSF melting curves of TS_VZV_ with BVDU_P_. (a) BVDU could not stabilize TS_VZV_ before phosphorylation but after an *in vitro* phosphorylation with TK_HS_, BVDU_P_ increased the T_m_ of TS_VZV_ by a larger extent than dUMP. (b) Varying concentrations of BVDU_P_ increased the T_m_ of TS_VZV_ in a dose responsive manner. (c) Further stabilization of TS_VZV_ was observed in the presence of 100 μM raltitrexed with 100 μM of BVDU_P_.

We were also interested in whether BVDU, a nucleoside based VZV drug with some similarities to the substrate dUMP, could also bind TS_VZV_. The binding of TS_VZV_ to BVDU before (BVDU) and after *in vitro* phosphorylation (BVDU_P_) with TK_HS_ was therefore studied with DSF. The addition of 100 μM BVDU to TS_VZV_ produced a dual-transition melting curve that is similar to the ligand free TS_VZV_ (Fig 3a). On the contrary, BVDU_P_ produced a single melting transition with a T_m_ of approximately 57°C in DSF. This observed stabilization of TS_VZV_ by BVDU_P_ is approximately 7°C higher than the stabilization of TS_VZV_ by 100 μM dUMP (Fig 3a). Varying concentrations of BVDU_P_ produced a dose responsive increment in the T_m_ of TS_VZV_ (Fig 3b). When 100 μM of raltitrexed was added to TS_VZV_ and 100 μM of BVDU_P_, the T_m_ of TS_VZV_ increased from 57°C to 64°C (Fig 3c).

## Discussion

TS is a highly conserved enzyme in many organisms including human, mouse, bacteria, protozoa and some viruses. TS is essential for the *de novo* synthesis of dTMP and its expression is up-regulated in actively replicating cells. It is an attractive drug target and several TS inhibitors including 5-fluorouracil, raltitrexed and pemetrexed are extensively used for treatments of solid tumors [39, 40]. Structural information of TS from several different species in complex with its substrates and inhibitors is also available [17–30]. TS is seemingly only present in two human herpesviruses, VZV and KSHV [32, 33]. Both TS_VZV_ and TS_KSHV_ share high sequence similarities with TS_HS_, with at least 80% sequence identity at the TS active site. Enzymatic studies have showed that TS_VZV_ and TS_KSHV_ display similar catalytic kinetics as human TS in the presence of dUMP and mTHF [32]. However, both herpesviral TS remain poorly characterized till date.

In an attempt to further characterize TS_VZV_, the crystal structures of the apo-, dUMP and BVDU_P_ bound TS_VZV_ were determined. These constitute the first crystal structure of an eukaryotic viral TS. The overall structure is, as expected, well conserved with the human TS (S1 Fig) [35]. However, the TS_VZV_+dUMP and TS_VZV_+BVDU_P_ structures have disordered regions that cannot be modeled in two of the four subunits of the crystallographic asymmetric unit. It is tempting to speculate that the disorder is correlated to the binding of the dUMP/BVDU_P_. However, these disordered regions are observed only in one of the monomer of the TS_VZV_ dimer. Although the binding of dUMP and BVDU_P_ appears to induce minor conformational changes to the amino acids lining the active site, the positioning of both dUMP/BVDU_P_ and the amino acids lining the active site are largely conserved in all the monomers (Fig 1). An inspection of the crystal packing in the unit cell of the TS_VZV_+dUMP and TS_VZV_+BVDU_P_ structures reveals that these disordered regions are located on the protein surfaces that lack crystal contact with a neighboring monomer. Thus, the observed structural disorder is most likely due to differences in crystal packing and crystal contacts of the two molecules and not due to the binding of dUMP/BVDU_P_ to TS_VZV_.

The TS_VZV_+dUMP structure adopts an open conformation, as concluded from the lack of covalent bonds between the thiol group of the catalytic cysteine and C6 of the dUMP pyrimidine ring (Fig 1b). The presence of the cofactor and the C-terminal loop have been proposed to be required for the closed conformation [41]. However, the TS_VZV_ protein construct that produced well diffracting crystals for structural determination of TS_VZV_ lacks the last six amino acids at the C-terminal. These six amino acids correspond to the C-terminal loop that has been reported to act as a lid over the TS active site and assist in folate binding [22]. Thus, the reason for an open conformation in TS_vzv_+dUMP is likely partly due to the absence of the folate cofactor in the active site and the C-terminal loop in the TS_VZV_ structures.

The interactions between dUMP/BVDU_P_ and an anti-folate drug raltitrexed with TS_VZV_ were characterized with DSF. While the DSF demonstrated that dUMP/BVDU_P_ and raltitrexed are able to bind to TS_VZV_ *in vitro*, it also showed that raltitrexed is not able to bind TS_VZV_ in the absence of the nucleotides (Figs 2c and 3c). This is consistent with the well-characterized sequential binding mode in other TSs [18]. Thus, the lack of binding of raltitrexed to TS_VZV_ in the DSF is likely due to the requirement for bound dUMP in the active site before raltitrexed can bind.

Nucleoside analogs have routinely been used for treatments of viral infections [7, 42]. Many of these drugs are administered as a pro-drug and requires an activation step through phosphorylation by the viral or human TK [43]. To study the possibility of TS_VZV_ being a target protein of nucleoside analogs, which are often not commercially available in their phosphorylated form, *in vitro* phosphorylation by TK_HS_ was performed prior to the binding studies. The assay was tested with deoxythymidine, which should be phosphorylated by TK_HS_ to TMP in the presence of ATP. The *in vitro* phosphorylation was validated with DSF, as deoxythymidine is unable to bind to TS_VZV_ while dTMP gave significant shifts (S2 Fig).

Several clinically approved anti-herpesviral nucleoside analogs are 5-substituted pyrimidine nucleosides, which are modifications of the TS binding dUMP. BVDU is a clinically approved 5-substituted pyrimidine nucleoside analog that displays the highest potency against HSV-1 and VZV [42, 44]. It has also been shown to be an inhibitor of TS_HS_ [45]. Our DSF experiments demonstrated that BVDU is able to bind TS_VZV_ after *in vitro* phosphorylation by TK_HS_ (Fig 3a). This supports that the nucleoside analog has been successfully phosphorylated by TK_HS_ *in vitro*. The successful phosphorylation of BVDU_P_ was eventually confirmed by the presence of the phosphate group on BVDU_P_ in the complex structure of TS_VZV_ with BVDU_P_ (Fig 1d). This may seem contradicting to previous studies that have reported a selective phosphorylation of BVDU by the herpesviral TK but TK_HS_ has been shown to be catalytically active towards BVDU, albeit with low efficiency [10, 46]. The effectiveness of TK_HS_ on BVDU may be attributed to the higher concentration of the kinase used *in vitro* and the efficiency of TK_HS_ activity on BVDU is likely to diminish *in vivo*.

The binding of BVDU_P_ to TS_VZV_ was further confirmed with the complex structure, TS_vzv_+BVDU_p_. The coordination of BVDU_P_ is highly similar to the coordination of dUMP in TS_VZV_ (Fig 1b and 1d). Though BVDU_P_ is not covalently bound to the catalytic thiol, a comparison of the structure of TS_vzv_+BVDU_P_ with the closed TS_HS_ shows that the coordination of BVDU_P_ by TS_VZV_ is more conserved with the coordination of dUMP in the closed-TS_HS_ (Fig 1c and 1d). Even in the absence of the folate cofactor and the C-terminal loop, BVDU_P_ appears to be held in a closed conformation by the additional hydrophobic interactions contributed by its 5-bromovinyl substituent (Fig 1e). The presence of a bound PEG 400 in the TS_VZV_ active site, in the region where folate binds, may have also helped in stabilizing the closed conformation. However, rather than a catalytically active closed conformation, the BVDU_P_ bound TS_VZV_ structure is likely to represent a catalytically inhibited TS_VZV_. Together, these data suggest that phosphorylated BVDU inhibits TS_VZV_ by binding competitively to the active site and yield further support for that TS_VZV_ could be a target protein for BVDU in the cell after the activation by the herpesvirus TK.

BVDU_P_ has been previously demonstrated to inhibit the catalytic activities of both TS_VZV_ and TS_HS_ *in vitro* [32, 47]. The high degree of structural conservation and the lack of selectivity of BVDU_P_ between TS_VZV_ and TS_HS_ could potentially lead to TS_HS_ mediated adverse effects. However, structural differences observed between TS_VZV_ and TS_HS_ may confer additional selectivity of BVDU_P_ towards TS_VZV_. His 184 do not share the same conformation as the corresponding His 196 in TS_HS_ (Fig 1c and 1d). As a result, the imidazole ring of His 184 does not interact with O4 of the dUMP/BVDU_P_ pyrimidine ring. Even though the amino acids lining the active sites of TS_VZV_ and TS_HS_ are highly conserved, there is a single amino acid substitution at Tyr 100 in TS_VZV_ that corresponds to Asn 112 in TS_HS_. This could potentially aid in the design of more specific inhibitors towards TS_VZV_. Regardless of the structural differences between TS_VZV_ and TS_HS_, the potency of BVDU towards VZV infected cells is likely due to the preferential phosphorylation by herpesvirus TK [10, 46]. Nevertheless, further studies are required to evaluate whether TS_VZV_ is indeed a physiological relevant target protein for the mono-phosphorylated BVDU.

In conclusion, substrate binding in TS_VZV_ has been characterized with a thermal shift assay and the apo and complex structures with nucleotides have been determined. The different TS_VZV_ structures share features with the evolutionary conserved TS_HS_ but also show some small but significant differences. Binding between TS_VZV_ and an activated herpesviral drug BVDU was demonstrated and a complex structure with an *in vitro* phosphorylated BVDU was determined. Together this work adds further support BVDU as a good inhibitor for TS_VZV_ and provides the structural basis for structure driven design of more specific TS_VZV_ inhibitors.

## Materials and Methods

### Cell culture and harvest

The starter culture supplemented with 50 μg/mL kanamycin (Sigma Aldrich) and 34 μg/mL chloroamphenicol (Sigma Aldrich) was inoculated with a streak from a TS_VZV_ glycerol stock. The culture was incubated overnight at 37°C, 220 rpm. 2 mL of the starter culture was added into each flask containing 750 mL of fresh TB, 50 μg/mL kanamycin and 34 μg/mL chloroamphenicol. The cultures were incubated at 37°C, 180 rpm until OD_600_ was 0.8. Expression was induced overnight with 0.5 mM IPTG at 18°C and harvested by centrifugation (4500 g for 10 minutes at 15°C) the next day. The cell pellet was flash frozen with liquid nitrogen, pounded and stored at -20°C.

### Protein purification

TS_VZV_ was purified with a two-step standard purification protocol, consisting of an immobilized metal affinity chromatography step and a size exclusion chromatography step using the ÄKTAxpress protein purification systems at 4°C. Ice-cold lysis buffer (100 mM Na-HEPES pH 8.0 (Sigma Aldrich), 500 mM NaCl (Sigma Aldrich), 10 mM imidazole pH 8.0 (Sigma Aldrich), 10% glycerol (Sigma Aldrich) and 0.5 mM TCEP (Sigma Aldrich)) supplemented with 0.1 mg/mL lysozyme (Sigma Aldrich), 1 μL/mL protease inhibitor cocktail (Nacalai) and 125 U/mL benzonase (Merck Millipore) was added to the frozen TS_VZV_ cell pellet. The cell lysate was sonicated before clarifying by centrifugation at 47000 g for 25 minutes at 4°C and filtration of the supernatant with an 1.2 μm syringe filter. The filtrate was loaded onto a pre-equilibrated (10 mM Na-HEPES pH 7.5, 500 mM NaCl, 10 mM imidazole pH 7.5, 10% glycerol and 0.5 mM TCEP) 5 mL HiTrap IMAC HP column (GE Healthcare) and the column was washed with 25 mL of purification wash buffer (10 mM Na-HEPES pH 7.5, 500 mM NaCl, 10 mM imidazole pH 7.5, 10% glycerol). The protein was eluted with a step elution of 156 mM and 335 mM imidazole. The samples eluted with 335 mM imidazole were pooled and concentrated to 5 mL before loading onto a pre-equilibrated (20 mM Na-HEPES pH 7.5, 300 mM NaCl, 10% glycerol and 0.5 mM TCEP) HiLoad 16/600 Superdex 200 gel filtration column (GE Healthcare). The fractions were analyzed on a 4–12% NuPAGE Bis-Tris gel (Life Technologies) and the purest fractions were pooled and concentrated to 12 mg/mL with a 10 kD MWCO protein concentrator (GE Healthcare). The concentrated TS_VZV_ samples were flash frozen in liquid nitrogen and stored at -80°C.

### *In vitro* phosphorylation of nucleosides using TK_HS_

Deoxythymidine was phosphorylated by incubating 1 mM deoxythymidine (Sigma-Aldrich) with 1 mM ATP (Sigma-Aldrich) and 40 μg of recombinant purified TK_HS_ (gift from Chen Dan, Nanyang Technological University, Singapore) overnight at 37°C in a reaction buffer (20 mM HEPES pH 7.5, 300 mM NaCl, 25 mM MgCl_2_). The reaction was stopped by heating the samples at 95°C for 15 minutes. The denatured TK_HS_ was removed by centrifugation at 13000 rpm for 5 minutes. The supernatant was removed and used for the crystallization and differential scanning fluorimetry (DSF) experiments with TS_VZV_. The phosphorylated BVDU (BVDU_P_) was prepared by incubating BVDU (Santa Cruz Biotechnology) with TK_HS_ as with deoxythymidine.

### Crystallization and data collection

The apo-TS_VZV_ was crystallized in two crystal forms. The first crystal form was obtained by mixing 200 nL of the crystallization solution (12% Ethylene glycol, 0.1 M HEPES pH 7.5) with 100 nL of the protein solution (12 mg/mL) in a 96-wells sitting drop Intelli-plate (Art Robbins) at 20°C. The second crystal form was obtained by adding 200 nL of the crystallization solution (0.1 M Tris-HCl pH 8.0 and 40% PEG 300) to 100 nL of the protein solution (12 mg/mL) in a 96-wells sitting drop Intelli-plate at 20°C. Crystals of the second crystal form were soaked with 1 mM dUMP for three minutes and 1 mM BVDU_P_ for six minutes for the structures of the complex of TS_VZV_ with dUMP (TS_VZV_+dUMP) and TS_VZV_ with BVDU_P_ (TS_VZV_+BVDU_P_) respectively. The crystals were then flash frozen with liquid nitrogen. Diffraction data were collected at the MX2 Micro Crystallography beam line at the Australian Synchrotron, Australia using the Blu-Ice data collection software [48]. All diffraction datasets were integrated and scaled with HKL2000 [49].

### Structure determination

The apo structure of TS_VZV_ was determined by molecular replacement with Phaser, using a molecule of TS_HS_ (PDB ID: 1HZW) as the search model [35]. Inspection of the electron density maps revealed spherical positive electron densities at one end of the TS_VZV_ active site and a phosphate ion was manually modeled into the positive electron densities using coot [50]. The structure of TS_VZV_ with phosphate was further refined with phenix.refine from the Phenix suite [51, 52].

The structures of TS_VZV_ in complex with dUMP (TS_VZV_+dUMP) and BVDU_P_ (TS_VZV_+BVDU_P_) were phased with molecular replacement using Phaser from the CCP4 suite [53, 54]. A TS_VZV_ monomer from the apo-TS_VZV_ structure was used as the molecular replacement search model. Inspection of the electron density maps of TS_VZV_+dUMP and TS_VZV_+BVDU_P_ revealed clear electron densities for dUMP and BVDU_P_ at their respective TS_VZV_ active sites. dUMP and BVDU_P_ were manually modeled into the respective positive electron densities using coot [50]. Iterations of automated and manual refinements of the structure were performed with phenix.refine and coot [50, 52]. The final structure refinement of the TS_VZV_ structures were validated with MolProbity [55]. All figures of the TS_VZV_ structures were displayed with pymol [56]. The apo TS_VZV_, TS_VZV_+dUMP and TS_VZV_+BVDU_P_ structures were deposited on the PDB as 4XSE, 4XSD and 4XSC.

### Differential scanning fluorimetry

TS_VZV_ was diluted to 0.2 mg/mL with the DSF buffer (20 mM HEPES pH 7.5 and 300 mM NaCl) supplemented with 5x SYPROrange (Life Technologies) and was added to the 96-wells PCR plate. Triplicates were performed. Samples were heated from 25°C to 80°C, in increments of 1°C. The average absolute fluorescence intensity of the triplicates was plotted against the temperature (°C) on Prism 6 (GraphPad, Prism). The curves were then normalized with Prism 6 to obtain the DSF melting curves of TS_VZV_. The melting temperatures (T_m_) of TS_VZV_ were estimated from the mid-point of each sigmoidal curve.

The effect of different ligands on TS_VZV_ was also performed with DSF as above, with slight modifications. In addition to diluting TS_VZV_ in the DSF buffer, TS_VZV_ was mixed with 1 mM dUMP (Sigma-Aldrich), 1 mM dTMP (Sigma-Aldrich), 1 mM deoxythymidine (Sigma-Aldrich) before and after phosphorylation or 1 mM BVDU (Santa Cruz) before and after phosphorylation. In the binding studies of raltitrexed with TS_VZV_, the DSF experiment was repeated with 100 μM of raltitrexed (Selleck Chemicals) in the presence and absence of 100 μM dUMP or BVDU_P_. The dose responsive DSF of TS_VZV_ with dUMP was obtained by mixing TS_VZV_ with dUMP at varying concentrations of 1 mM, 200 μM, 100 μM, 50 μM, 25 μM, 12.5 μM, 6.25 μM, 3.125 μM, 1.56 μM, 0.78 μM and 0 μM. Likewise, the dose responsive DSF of TS_VZV_ with the phosphorylated BVDU was also performed by mixing TS_VZV_ with 625 μM, 313 μM, 156 μM, 78 μM and 39 μM of BVDU_P_. The normalized melting curves and the T_m_ of TS_VZV_ in the presence of different ligands were obtained as described above.

## Supporting Information

S1 Fig Crystal structure of apo-TS_VZV_. Two orientations of the apo-TS_VZV_ dimer are illustrated in cartoon. A phosphate ion has been found in each TS_VZV_ active site and both phosphate ions are displayed as sticks.
(TIF)

S2 Fig DSF melting curves of TS_VZV_ with deoxythymidine before and after phosphorylation. Deoxythymidine did not stabilize TS_VZV_ before phosphorylation but after an *in vitro* phosphorylation with TK_HS_, it stabilizes TS_VZV_ to a similar extent as dTMP.
(TIF)
