# Supplementary figures and images for: Structure of the Varicella Zoster Virus Thymidylate Synthase Establishes Functional and Structural Similarities as the Human Enzyme and Potentiates Itself as a Target of Brivudine
Source: PLoS One. 2015 Dec 2;10(12):e0143947. doi: 10.1371/journal.pone.0143947 (PMC4668047; doi:10.1371/journal.pone.0143947)

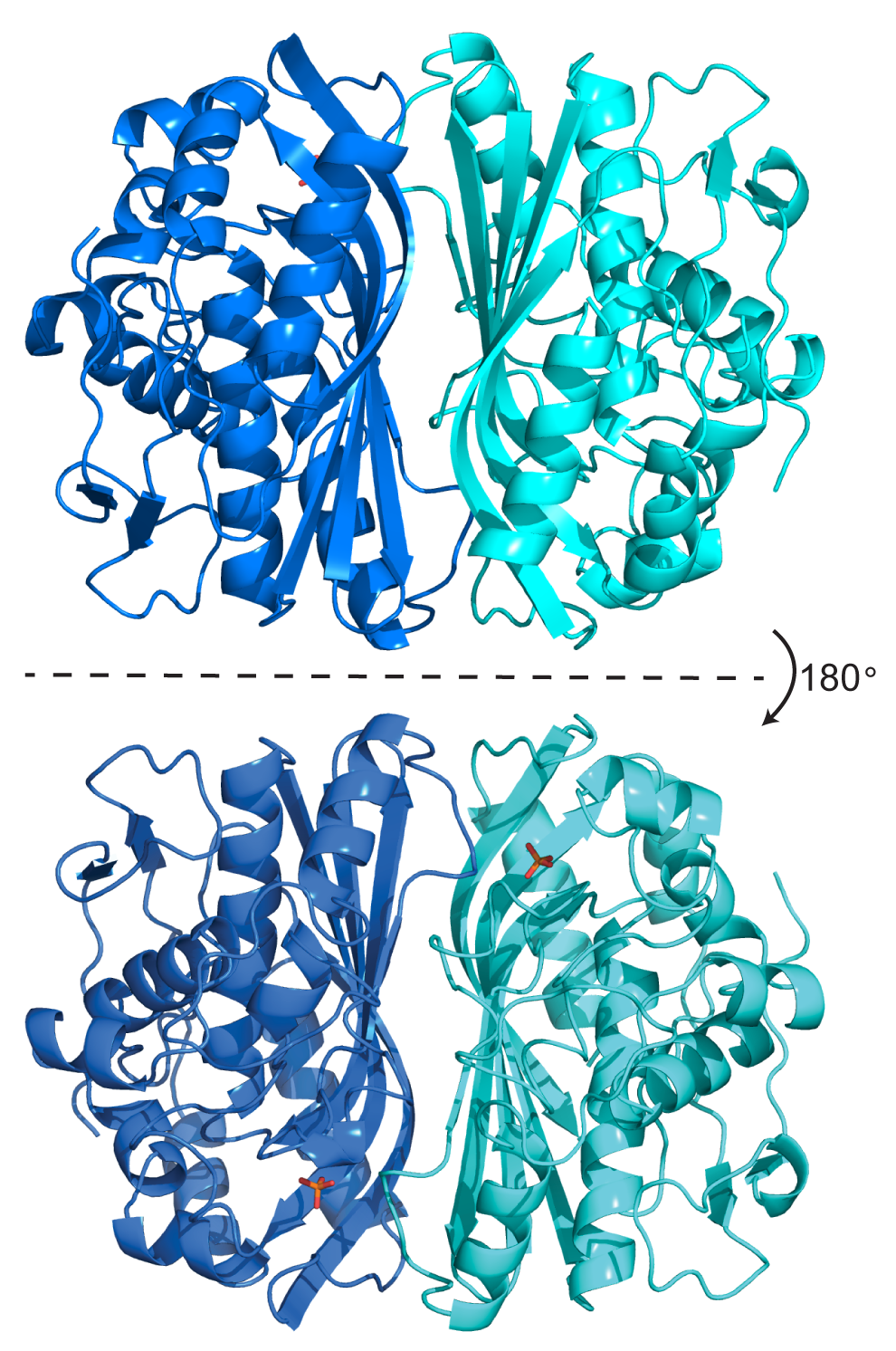

Supplement: S1 Fig — Two orientations of the apo-TSVZV dimer are illustrated in cartoon. A phosphate ion has been found in each TSVZV active site and both phosphate ions are displayed as sticks. (TIF) [file pone.0143947.s001.tif]

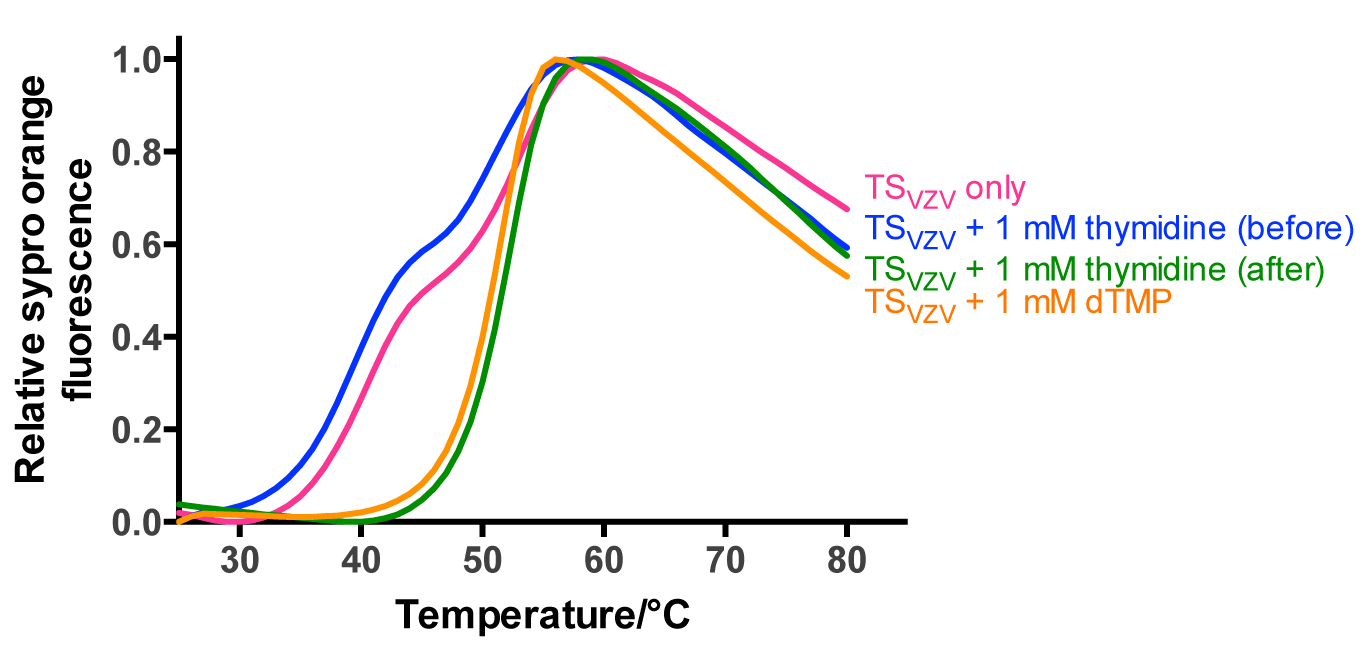

Supplement: S2 Fig — Deoxythymidine did not stabilize TSVZV before phosphorylation but after an in vitro phosphorylation with TKHS, it stabilizes TSVZV to a similar extent as dTMP. (TIF) [file pone.0143947.s002.tif]
